# Chiral Luminescent Liquid Crystal with Multi‐State‐Reversibility: Breakthrough in Advanced Anti‐Counterfeiting Materials

**DOI:** 10.1002/advs.202201565

**Published:** 2022-05-01

**Authors:** Yonghong Shi, Jianlei Han, Xue Jin, Wangen Miao, Yi Zhang, Pengfei Duan

**Affiliations:** ^1^ CAS Key Laboratory of Nanosystem and Hierarchical Fabrication National Center for Nanoscience and Technology (NCNST) No. 11 ZhongGuanCun BeiYiTiao Beijing 100190 P. R. China; ^2^ University of Chinese Academy of Sciences Beijing 100049 P. R. China; ^3^ Chemistry and Chemical Engineering Institute of Physical Chemistry Lingnan Normal University Zhanjiang 524048 P. R. China; ^4^ Hefei BOE Display Technology Co. Ltd. No. 3166 Tonglingbei Road Hefei 230011 P. R. China

**Keywords:** anti‐counterfeiting, chirality, circularly polarized luminescence, energy transfer, spiropyran

## Abstract

Creating a security material that carries distinct information in reflective color, fluorescence, and chiroptical property will enhance anti‐counterfeiting levels to deter counterfeits ranging from currencies to pharmaceuticals, but is proven extremely challenging. In this work, an advanced anti‐counterfeiting material, with three‐state of each mode reversibly converted into multi‐mode materials including reflective color, fluorescence, and circularly polarized luminescence signal, is constructed by loading photofluorochromic spiropyran (SP) and zinc ion (Zn^2+^) into the chiral liquid crystal. Under UV irradiation, the complexes of SP and Zn^2+^ will be transformed into merocyanine (MC) and MC‐Zn^2+^, while the energy transfer occurs from MC‐Zn^2+^ to MC. Upon heating, MC is easy to recover to SP, while the MC‐Zn^2+^ remains unchanged. The MC and MC‐Zn^2+^ can be transformed into the SP and Zn^2+^ under visible light irradiation. The three states of each mode can reversibly convert. Furthermore, the reflective color or fluorescence of each state shows different intensities under left‐ and right‐handed circular polarized filters, enabling easy distinguishing by naked eyes. The advanced anti‐counterfeiting method with multi‐state of each mode for multi‐mode encryption information output will provide a new concept for designing and fabricating multi‐mode anti‐counterfeiting materials, improving the security level for practical application.

## Graphical abstract

Advanced anti‐counterfeiting material has multi‐mode information outputs including reflective color, fluorescence, and circularly polarized luminescence signal. Each mode has three states, and the three‐state information can be reversibly converted. Assisted by the left‐ and right‐handed circular polarized filters, the intensity of reflective color and fluorescence of each state can be distinguished by the naked eye.

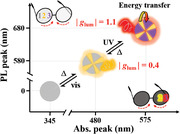

## 1 Introduction

The development of anti‐counterfeiting materials has attracted particular attention in order to prevent the increasing counterfeits on the current market, including brands, luxury items, banknotes, tickets, and certificates.^[^ ^1^, ^2^, ^3^, ^4^, ^5^, ^6^, ^7^, ^8^, ^9^ ^]^ In the past few decades, a wide variety of security and anti‐counterfeiting technologies have been developed, including thermal,^[^ ^10^, ^11^, ^12^, ^13^ ^]^ magnetic,^[^ ^14^ ^]^ light,^[^ ^15^, ^16^, ^17^ ^]^ and mechanical responses.^[^ ^18^, ^19^ ^]^ Therein, the single‐mode (photoluminescence or reflective color) with single‐state (only one reflective color or fluorescence) is the most widely used, possessing the advantages of a broad range of materials sources, easy operation, and observation.^[^ ^20^, ^21^, ^22^ ^]^ Typically, the traditional single‐state photoluminescence generally emits a single‐color fluorescence excited by a single light source.^[^ ^23^, ^24^ ^]^ Moreover, single‐mode with multi‐states fluorescence (different colors of fluorescence) has also been developed by mixing several organic or inorganic materials.^[^ ^25^, ^26^ ^]^ When the excitation wavelength changes, the materials show excitation‐wavelength‐dependent fluorescence colors. Unfortunately, the application of these technologies with single‐ or multi‐state in single‐mode is relatively ineffective and easy to crack. Therefore, it is very important to develop more hidden and multi‐dimensional technologies for anti‐counterfeiting applications.

**Figure 1:**
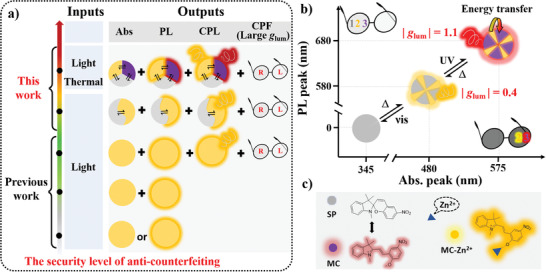
a) The diagram of the security level of anti‐counterfeiting. When selective different inputs (light or thermal), corresponding modes will be the outputs, such as reflective color, fluorescence, and CPL signal, where each mode can be divided into single‐state or multi‐state. In the case of multi‐state in each mode, they can be reversibly converted. The glasses consisting of L‐CPF and R‐CPF were placed on the sample with large g_lum_ values and significant differences could be observed by the naked eye. b) Advanced anti‐counterfeiting materials shows multi‐mode characteristic with reflective color, fluorescence, and CPL signal, where each mode has three states, and three states can be reversibly converted; the insets show photographs of each state by the glasses with L‐CPF and R‐CPF (top‐left corner: natural light; lower‐right corner: under 365 nm light). c) Molecular structures of three states used for anti‐counterfeiting.

Chiral functional materials with a large luminescence dissymmetry factor (g_lum_) have risen to prominence in recent years due to their fascinating multi‐mode optical properties (such as fluorescence or circularly polarized luminescence [CPL]) and hidden information in anti‐counterfeiting technology.^[^ ^27^, ^28^ ^]^ Meaningfully, the CPL materials with a large *g*_lum_ value show different fluorescence intensity under the left‐ and right‐handed circular polarized filter (L‐CPF and R‐CPF).^[^ ^27^, ^29^, ^30^ ^]^ However, the obtained *g*_lum_ value of the general CPL materials is very low.^[^ ^31^, ^32^, ^33^, ^34^, ^35^, ^36^ ^]^ So far, the chiral liquid crystals (N*LC) are recognized as the most effective matrix for fabricating the CPL‐active materials with a large *g*_lum_ value.^[^ ^30^, ^37^, ^38^ ^]^ Particularly, the N*LC system can be prepared by simply doping the chiral compounds into the achiral nematic liquid crystal. To obtain the CPL‐active materials with a large *g*_lum_ value, the emission of doped emitters is generally located in the center of the photonic bandgap.^[^ ^37^, ^39^, ^40^ ^]^ By changing the ratio of chiral dopants, the photonic bandgap of N*LC could be flexibly tuned. Cheng's group has synthesized binaphthyl enantiomers as chiral dopants and introduced four aggregation induced emission dyes into cholesteric liquid crystals, which can produce strong CPL with |*g*_lum_| values of up to 1.42.^[^ ^41^ ^]^ However, most of the reported cases have been limited to single‐state of dual‐mode (**Figure** **1**) or relatively small *g*_lum_ value, which restricts the security level of anti‐counterfeiting techniques.

To obtain a high level of anti‐counterfeiting performance, it is very necessary to design materials with multi‐mode characteristics, such as reflective color, fluorescence, and CPL signal, where each mode not only has multi‐states but also the multi‐states can be reversibly converted (Figure 1). Thus, the researchers pay more attention to stimuli‐responsive materials, particularly light‐responsive materials such as spiropyran (SP),^[^ ^42^, ^43^, ^44^, ^45^, ^46^, ^47^, ^48^, ^49^ ^]^ diarylethene,^[^ ^50^, ^51^, ^52^ ^]^ and azobenzene.^[^ ^53^, ^54^ ^]^ These stimuli‐responsive materials will exhibit dynamic changes of reflective color or fluorescence over time through external stimuli, including heat, irradiation, mechanical force, and electromagnetic field.^[^ ^55^, ^56^, ^57^ ^]^ Yang et al. have reported a light‐driven cholesteric liquid crystal, which was composed of light‐driven chiral molecular motor (working as chiral dopant) and fluorescent dyes.^[^ ^27^ ^]^ The reflection band and CPL intensity of the sample can be regulated by UV 365 nm irradiation. Moreover, when an L‐CPF or R‐CPF is placed on the sample, an obvious differential could be seen whether under the daylight or UV light. However, realizing the multi‐reflective color, multi‐fluorescence, and variable CPL signal requires the replacement of fluorescence dyes, which limits their practical application. The spiropyran are well‐known photochromic compounds, that is, undergoing reversible structural isomerization between a colorless SP form (non‐photoluminescent) and a purple‐colored at merocyanine form (MC, photoluminescent) by UV light and vice versa by visible light or heat. Interestingly, when incorporated Zn^2+^ into the MC system, MC may become thermodynamically favorable, enabling thermally‐triggered isomerization of SP to the complexes of merocyanine and zinc ion (MC‐Zn^2+^).^[^ ^58^, ^59^, ^60^, ^61^ ^]^ Meanwhile, the absorption and emission spectrum of MC‐Zn^2+^ is very different from that of MC form, and there may be energy transfer between them due to the matchable emission spectrum of MC‐Zn^2+^ and the absorption spectrum of MC. Additionally, the metal ion from MC‐Zn^2+^ can be released by visible light irradiation (Figure 1). Inspired by the multi‐stimuli‐responsive nature and the possible energy transfer property, we skillfully designed a kind of spiropyran‐based materials that exhibit multi‐mode characteristics with the reversible multi‐state responsiveness for each mode under the external stimulation (365 or 465 nm or heat). Considering that the reversible conversion between SP and MC is too fast in solution and too slow in the solid state, it is not easy to operate in practical applications. Therefore, we choose liquid crystal as an ideal medium, which can provide an excellent environment for stimulus‐responsive materials, enabling them to flexibly regulate their switching rate. More important, chiral liquid crystals can endow emitters with extremely large *g*_lum_ value.

In this work, we present promising multi‐mode anti‐counterfeiting materials for application in security labels and encoding encryption, which are fabricated by dispersing excess photofluorochromic SP and a small amount of Zn^2+^ into N*LC (Figure 1). Under UV light, MC and MC‐Zn^2+^ coexist, while the energy transfer will occur from MC‐Zn^2+^ to MC isomer. The MC/MC‐Zn^2+^‐N*LC system shows purple color, while intense red CPL emission with |*g*_lum_| value up to 1.1 at 685 nm can be observed. Subsequently, upon heating (≈50 °C), MC is quickly converted to an SP isomer, while the MC‐Zn^2+^ isomer remains unchanged. The orange color SP/MC‐Zn^2+^‐N*LC exhibits good CPL performance with the |*g*_lum_| value up to 0.4 at 590 nm. Finally, MC and MC‐Zn^2+^ can be easily converted to colorless SP, releasing Zn^2+^ (SP/Zn^2+^‐N*LC) under visible light irradiation. Compared with previously reported multi‐mode materials, the system has three advantages as anti‐counterfeiting materials. First, the system has wide materials sources and does not need tedious synthesis. Second, three states of each mode in a multi‐mode system present reversible conversion by light or heat stimulation. Each state could exhibit dynamic changes of reflective color and fluorescence color with high contrast. Third, owing to the large *g*_lum_ value of the multi‐mode system, a striking distinction of reflective color and fluorescence of each state can be observed assisted by L‐CPF and R‐CPF. The studies open novel venues for technological advances in anti‐counterfeiting research, and the multi‐mode stimuli‐responsive materials coupled with N*LC enable rational design and scalable production of advanced anti‐counterfeiting materials.

## 2 Results and Discussion

**Figure 2:**
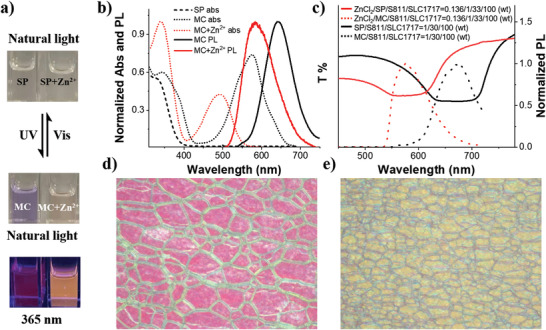
a) Photographs show the interconversion of reflective color and fluorescence of sample by external stimulation (UV 365 and 465 nm light) between SP and MC, and between SP‐Zn^2+^ and MC‐Zn^2+^, the concentration of the sample is about 5 × 10^−5^ m in ethyl acetate. b) Normalized absorption and fluorescence spectra of SP, MC, and MC‐Zn^2+^ in ethyl acetate (SP = MC = MC‐Zn^2+^ = 5 × 10^−5^ m, *λ*_ex_ = 360 nm). c) Transmittances (SP and SP/Zn^2+^) and emission (MC and MC‐Zn^2+^) spectra of photofluorochromic isomers in the N*LC. The weight ratios of SP/*S*811/SLC1717 = 1/30/100, the weight ratios of ZnCl_2_/SP/ *S*811/SLC1717 = 0.136/1/33/100. Polarized optical microscopy (POM) images of d) SP‐N*LC and e) SP/Zn^2+^‐N*LC.

In this work, an advanced anti‐counterfeiting material is constructed by loading a photofluorochromic SP, Zn^2+^, and chiral *R*811(or *S*811) into the liquid crystal SLC1717 (Figure S1, Supporting Information). The results show that the materials possess multi‐mode outputs of reflective color, fluorescence, and CPL signal, with three states for each mode, while the three states can be reversibly converted (Figure 1). Upon exposure to UV 365 nm light, the ring‐closed form SP (colorless, nonluminous) can reversibly isomerize to the ring‐opened form MC (purple‐colored) with induced photoluminescence (650 nm) in ethyl acetate (**Figure** **2**a,b). In addition, the MC has a phenolate group, which can readily bind with zinc ions (MC‐Zn^2+^). The MC‐Zn^2+^ has an absorption peak at 480 nm with an orange emission color located at ≈580 nm. The emission of MC‐Zn^2+^ shows a blue‐shift relative to the MC isomer, and the visual fluorescence color changes from red to yellow after coordination with Zn^2+^ (Figure 2a and Figure S2, Supporting Information). Under visible light, the MC and MC‐Zn^2+^ isomer can be easily converted to initial SP and SP/Zn^2+^. The photoswitching reaction of MC and MC‐Zn^2+^ was conducted repeatedly by alternating the irradiation of UV 365 and 465 nm light. There was no significant bleaching in emission intensity after ten cycles, indicating an excellent fatigue resistance (Figure S3, Supporting Information). Moreover, the coordination between Zn^2+^ and MC can significantly suppress the spontaneous transformation from MC to SP isomer (Figure S4, Supporting Information).

To simultaneously obtain the best reflective color, fluorescence intensity, and CPL performance, the optimum ratio of the components in N*LC was thoroughly investigated. First of all, various amounts of SP isomer were dispersed into SLC1717 and were determined by polarizing optical microscopy (POM) (Figure S5, Supporting Information). It can be observed that SP aggregates when the weight ratio of SP/SLC1717 exceeds 1 wt%. In addition, as the content of MC increased, the absorption intensity increased (*λ*_abs_ = 573 nm), and the emission peak showed a red‐shift (Figure S6, Supporting Information). The photographs of MC/SLC1717 with different weight ratios are shown in Figure S7, Supporting Information, under visible light (or heat) and UV light. To obtain a high‐contrast reflective color without aggregation of SP, the mixing ratio of SP/SLC1717 at 1 wt% is confirmed to be the best. It has been demonstrated that locating the emission peak within the photonic bandgap of N*LC will enable the CPL emission with the highest *g*_lum_ value. Thus, after fixing the weight ratio of SP/SLC1717 at 1 wt%, we tuned the photonic bandgap by changing the amount of chiral dopant *S*811/SLC1717 from 28, 30, and 32 wt% to 33 wt% (Figure S8, Supporting Information). Figure 2c shows that the location of the emission peak of MC in 30 wt% *S*811/SLC1717 overlaps well with the photonic bandgap. Moreover, the MC and SP isomer of photofluorochromic does not affect the position of the photonic bandgap (Figure S9, Supporting Information). Subsequently, the photophysical properties of the MC‐Zn^2+^ isomer were investigated in N*LC. Different ratio of ZnCl_2_ was introduced into liquid crystal with the ZnCl_2_/SLC1717 ratio from 0.14, 0.27, 0.54, and 1.36 wt% to 2.72 wt% (SP/*S*811/SLC1717 = 1/33/100 wt%). The POM images (Figure S10, Supporting Information) suggested that when the ratio of ZnCl_2_/SLC1717 reached 2.72 wt%, an obvious aggregation phenomenon was observed. Meanwhile, the spectrum of absorption and emission of MC‐N*LC blue‐shifted by increasing the amount of ZnCl_2_ (Figure S11, Supporting Information). Corresponding photographs of ZnCl_2_/SLC1717 with different weight ratios in MC‐N*LC under irradiation with visible 465 nm light and UV 365 nm light are shown in Figure S12, Supporting Information. To obtain the best reflective color and fluorescence without aggregation, we fixed the ratio of ZnCl_2_/SLC1717 at 1.36 wt%, which is the optimal ratio in the MC‐N*LC system. Moreover, the emission peak of MC‐Zn^2+^‐N*LC (33 wt% in chiral liquid crystal *R*811/SLC1717) overlaps with the photonic bandgap (Figure 2c). Based on the results of POM measurements, we could clearly see that the planar texture of N*LC does not change even though photochromic and photofluorochromic reactions occurred (Figure 2d,e and Figure S13, Supporting Information).

**Figure 3:**
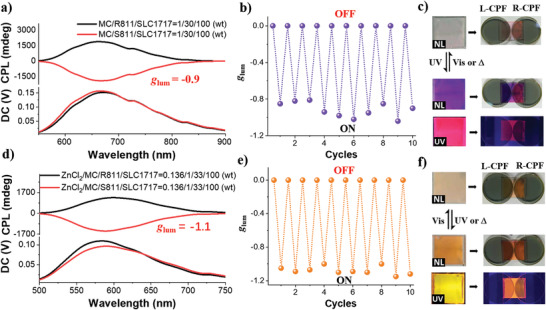
CPL spectra of a) MC‐N*LC and d) MC‐Zn^2+^‐N*LC excited at 390 nm. The weight ratios of MC/*R*811(*S*811)/SLC1717 = 1/30/100, the weight ratios of ZnCl_2_/MC/*R*811(*S*811)/SLC1717 = 0.136/1/33/100. The photoswitching CPL *g*_lum_ value of the b) MC‐N*LC and e) MC‐Zn^2+^‐N*LC monitored at 680 and 580 nm upon alternating UV light (365 nm) and visible light (465 nm) irradiation, respectively. The reversible conversion picture of the c) MC‐N*LC and f) MC‐Zn^2+^‐N*LC in natural light (NL) and 365 nm (UV) by the external stimulation. The corresponding pictures show that the intensity of reflective color and fluorescence have distinctly different when the L‐CPF and R‐CPF were placed on top of the sample.

Dynamic CPL systems with large *g*_lum_ values have attracted extensive attention because they not only have fluorescence switching characteristics but also can display different intensities of fluorescence under the L‐CPF and R‐CPF. The mirror‐imaged CPL signals of MC‐N*LC are shown in **Figure** **3**a, where the MC‐N*LC induced by *R*811 and *S*811 exhibited positive and negative CPL signals, respectively. These results suggested that chiral dopant *R*811 would induce the nematic liquid crystal to a right‐handed N*LC, while the addition of *S*811 to SLC1717 will result in a left‐handed N*LC. The corresponding |*g*_lum_| value was 0.9 at 680 nm. Additionally, the CPL emission could be switched ON/OFF by alternating the photoirradiation of 365 or 465 nm light (or heat) (Figure 3b).

In the initial state, a colorless SP in N*LC was obtained (Figure 3c). Upon exposure to UV 365 nm light, SP‐N*LC changed from colorless to purple color (MC‐N*LC) with red emission. Subsequently, the MC‐N*LC would return to the SP‐N*LC isomer via staying in dark or irradiating by visible light (465 nm light). Next, we placed the L‐CPF and R‐CPF on the surface of the sample, the reflective color obviously darkened when the L‐CPF covered the sample under natural light (NL) and UV 365 nm light. However, the sample was clearly observed by changing to R‐CPF. Moreover, under UV irradiation, the emission was more clearly observed through L‐CPF compared with the R‐CPF (Figures S14 and S15, Supporting Information). A similar phenomenon has been observed in the SP/Zn^2+^‐N*LC systems composed of both SP and Zn^2+^. Figure 3d shows the mirror‐imaged CPL signal of the MC‐Zn^2+^‐N*LC system, which showed a large |*g*_lum_| value of around 1.1 at 580 nm. Additionally, a light‐regulated CPL conversion was also realized with great fatigue resistance (Figure 3e).

Surprisingly, simultaneously introducing MC and Zn^2+^ into N*LC will significantly change the overall state of the complex N*LC. In the initial state, the colorless SP/Zn^2+^‐N*LC sample could turn to orange color (MC‐Zn^2+^‐N*LC) upon heating or irradiation with UV 365 nm light (Figure 3f), which was totally different from the SP‐N*LC system (Figure 3c). The reflective color and fluorescence of MC‐Zn^2+^‐N*LC also showed reversible conversion under alternating irradiation of UV 365 nm (or heat) and 465 nm light. The intensity of both reflective color and fluorescence color of the MC‐Zn^2+^‐N*LC showed obvious differential under the L‐CPF and R‐CPF (Figure 3f and Figure S14, Supporting Information). Moreover, we have analyzed the effect of the dopants on the liquid crystal behaviors by X‐ray diffraction (XRD) measurements. The XRD results indicated that all liquid crystal samples showed similar diffraction patterns compared to the original nematic liquid crystal SLC1717 (Figure S16, Supporting Information). It can be explained that the dopants do not interfere with N*LC.

From the normalized absorption and emission spectra of MC and MC‐Zn^2+^ in ethyl acetate, we can clearly see that the emission band of MC‐Zn^2+^ (580 nm) overlaps well with the absorption band of MC. Thus, by exciting the MC‐Zn^2+^, energy transfer may occur from MC‐Zn^2+^ to MC. Then, we verified the energy transfer process in ethyl acetate (Figure S17, Supporting Information). Gradually adding MC to MC‐Zn^2+^ by fixing the concentration of MC‐Zn^2+^ at 10^−5^ m would gradually decrease the emission intensity of MC‐Zn^2+^, while the emission intensity of MC increased. Similar results have been confirmed in the N*LC (Figure S18, Supporting Information). In addition, the emission decay of MC‐Zn^2+^ became 0.6 ns in the presence of MC (15 eq) compared to that of MC‐Zn^2+^ (1.1 ns) without MC (Figure S19, Supporting Information). The shortening of the emission decay of the MC‐Zn^2+^ in the presence of MC indicated that the Förster resonance energy transfer process might be the main mechanism for the energy transfer process.^[^ ^62^, ^63^ ^]^

Inspired by the multi‐stimuli‐responsive nature and energy transfer property, the multi‐mode materials containing MC and MC‐Zn^2+^ in N*LC were designed (Figure S20a–c, Supporting Information). The MC/MC‐Zn^2+^‐N*LC system was purple in color, while red fluorescence can be observed. Subsequently, upon heating, MC is quickly converted to an SP isomer, while the MC‐Zn^2+^ isomer remains unchanged. The SP/MC‐Zn^2+^‐N*LC system was in orange color with bright yellow fluorescence. The pictures of samples with different ratios of Zn^2+^ in MC/*S*811/SLC1717 under NL and UV 365 nm light are shown in Figure S12, Supporting Information. When the weight ratio of ZnCl_2_/MC/*S*811/SLC1717 is 0.027:1:30:100, we can clearly see that the reflective color and fluorescence of each state are different and highly distinguishable. We have measured the fluorescence spectra of the SP/MC‐Zn^2+^‐N*LC system under the stimulation of light and heat cycles, which further confirm that there is no isomerization of MC‐Zn^2+^ in the N*LC system upon heating (Figure S20d, Supporting Information). In addition, we can clearly see from the POM image that the conversion between each state does not affect the planar texture of the N*LC (Figure S21, Supporting Information).

**Figure 4:**
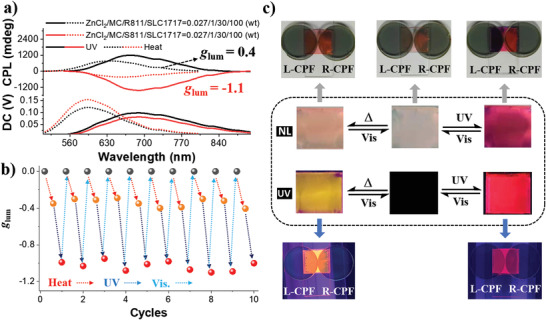
a) CPL spectra of multi‐states forms with SP/MC‐Zn^2+^ and MC/MC‐Zn^2+^ of N*LC sample under heat and light (*λ*_ex_ = 400 nm). The weight ratios of ZnCl_2_/MC/*R*811(*S*811)/SLC1717 = 0.027/1/30/100. b) The CPL *g*_lum_ value of the MC/MC‐Zn^2+^‐N*LC and SP/MC‐Zn^2+^‐N*LC monitored at 685 and 590 nm upon alternating heat, UV light (365 nm), and visible light (465 nm), respectively. c) The reversible conversion picture of the SP/Zn^2+^‐N*LC, SP/MC‐Zn^2+^‐N*LC, and MC/MC‐Zn^2+^‐N*LC in natural light (NL) and UV 365 nm by the external stimulation, and the outside corresponding picture under the L‐CPF and R‐CPF.

Subsequently, the multi‐mode N*LC, including reflective color, fluorescence, and CPL signal, were thoroughly investigated. Each mode has three states (SP/Zn^2+^, SP/MC‐Zn^2+^, and MC/MC‐Zn^2+^), which can be reversibly converted between each other. Upon irradiation with UV 365 nm light, the mirror‐imaged CPL signals of MC**/**MC‐Zn^2+^‐N*LC were observed at 685 nm (**Figure** **4**a). Since the emission peak was located at the center of the photonic bandgap, the sample emitted intense CPL with a large |*g*_lum_| value up to 1.1. Then, the emission peak of SP**/**MC‐Zn^2+^‐N*LC will shift to 590 nm by heating, and the mirror‐imaged CPL signals with a |*g*_lum_| value around 0.4 could be obtained. Meanwhile, the CPL signal of SP**/**MC‐Zn^2+^‐N*LC showed a slight red‐shift compared with the emission peak due to the effect of the photonic bandgap.^[^ ^64^ ^]^ Intriguingly, the CPL switches process was conducted repeatedly by alternating the external stimulation of UV 365 nm light, heat, and 465 nm light. There was no obvious attenuation of the CPL signal intensity after ten cycles (Figure 4b). We could directly see the change of reflective color and fluorescence during the photo‐ and thermo‐assisted isomerization (Figure 4c). The three states of reflective color (purple, orange, and colorless) and fluorescence (red, yellow, and non‐luminous) are reversibly converted under alternating UV 365 nm, heating, and visible light 465 nm. Meanwhile, the intensity of reflective color or fluorescence of each state is evidently different under the L‐CPF and R‐CPF.

**Figure 5:**
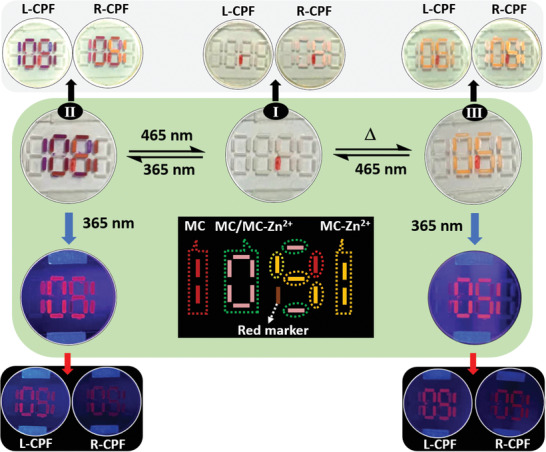
Advanced information encryption and decryption based on reflective color, fluorescence, and CPL, exhibiting a dynamic change in the reflective color and fluorescence. There are visual differences in the digital symbols, reflective color, and corresponding fluorescence in each state (I or II or III), and each state shows different intensity of reflective color and fluorescence assisted by the L‐CPF and R‐CPF, respectively. The variable digital codes in the middle of the picture (black background) contain useful code (MC, MC/MC‐Zn^2+^ and MC‐Zn^2+^) and fake code (red marker ink).

The multi‐mode materials can be further extended to the advanced anti‐counterfeiting field. As a proof‐of‐concept of enhanced anti‐counterfeiting level, an encrypted message pattern “1081” was created as schematically depicted in **Figure** **5** (dotted), where “1081” was constructed by MC, MC/MC‐Zn^2+^, MC‐Zn^2+^, and red marker. Since the red marker pen affects the reflective color other than the fluorescence, it has diversified signal outputs in reflective color and fluorescence. There is only a red vertical line in the initial state when the SP isomer and red marker line coexist (state‐I). Irradiated by UV 365 nm light, the reflective color of the encrypted message changes the red vertical line to “1081” composed of purple, red, and orange color (state‐II). Under UV 365 nm light, the corresponding encrypted message was shown as “1091” with different fluorescence colors. Then, upon heating, the MC isomer will quickly convert to an SP isomer in the encrypted message pattern, while the MC‐Zn^2+^ isomer remains unchanged. Meanwhile, the reflective color of the encrypted message pattern gave a “061” pattern (state‐III), the corresponding fluorescence pattern is “051” under UV 365 nm light with different fluorescence intensity. Finally, an encrypted message pattern can return to its initial state under 465 nm light irradiation (state‐I). More interestingly, the intensity of reflective color and fluorescence of each state is different under the L‐CPF and R‐CPF (Figure 5). To further confirm the generality of an anti‐counterfeiting pattern, we could make handy graphics freely. It can be seen that the shape of the pattern showed different reflective colors and fluorescence upon alternating stimulation of heat, UV (365 nm), and visible (465 nm) light (Figure S22, Supporting Information).

## 3 Conclusion

In summary, a multi‐mode material including reflective color, fluorescence, and CPL is designed for the advanced anti‐counterfeiting applications, where each mode has three states, while three states can be reversibly converted. Owing to the unique properties of both MC and MC‐Zn^2+^, the sample exhibited dynamic CPL performance from CPL‐silent to intense CPL emission (|*g*_lum_| = 1.1, at 685 nm) in response to UV irradiation based on the energy transfer process, which can convert to orange fluorescence (|*g*_lum_| = 0.4, at 590 nm) on heating. It can be recovered to its initial state when exposed to visible light. Except for fluorescence and CPL signal, the versatile reflective color of the sample can also be triggered by alternating stimulation of heat, UV, and visible light. More interestingly, the change in the intensity of reflective color and fluorescence of each state are easily distinguishable by naked eyes assisted by the L‐CPF and R‐CPF. Our strategy could provide a new concept for designing and fabricating multi‐mode anti‐counterfeiting materials possessing the capability of reversible multi‐states conversion, thus improving the security level for practical application.

## 4 Experimental Section

### Materials

Commercial room‐temperature nematic liquid crystal, SLC1717, was bought from the Chengzhi Yonghua Display Material Co., Ltd. Chiral dopant, *R(S)*811, was bought from the TOKYO Chemical Industry Co., Ltd. Zinc chloride (anhydrous, 98%) was purchased from innochem. The 4,4,5,5‐Tetramethyl‐2‐(10‐phenylanthracen‐9‐yl)‐1,3,2‐dioxaborolane (98%) was bought from the Bide Pharmatech Ltd. in shanghai.

### Characterizations

UV–vis and fluorescence spectra were recorded on the Hitachi U‐3900 spectrophotometer and F‐4500 fluorescence spectrophotometer, respectively. CPL spectra were measured on JASCO CPL‐200 spectrophotometers. Lifetime measurements were recorded on the spectrometer using time‐correlated single‐photon counting. POM images were recorded on Leica DM2700M upright materials microscope. XRD spectra were measured on the Rigaku D/Max‐2500 X‐ray diffractometer (Japan) with Cu/K*α* radiation (*λ* = 1.5406 Å). The photoreversion reactions were conducted by irradiation with 465 nm blue LED (HLV2‐ 22BL‐3W, CCS Inc.) or UV 365 nm.

## Conflict of Interest

The authors declare no conflict of interest.

## Supporting information

Supporting information

## Data Availability

The data that support the findings of this study are available from the corresponding author upon reasonable request.
