# Supplementary material for: Chiral Luminescent Liquid Crystal with Multi‐State‐Reversibility: Breakthrough in Advanced Anti‐Counterfeiting Materials
Source: Adv Sci (Weinh). 2022 May 1;9(20):2201565. doi: 10.1002/advs.202201565 (PMC9284135; doi:10.1002/advs.202201565)
Supplement: Supplementary file 1 — Supporting information [file ADVS-9-2201565-s001.pdf]

ADVANCED SCIENCE
Open Access

Supporting Information

for *Adv. Sci.*, DOI 10.1002/advs.202201565

Chiral Luminescent Liquid Crystal with Multi-State-Reversibility: Breakthrough in Advanced Anti-Counterfeiting Materials

*Yonghong Shi, Jianlei Han, Xue Jin, Wangen Miao, Yi Zhang and Pengfei Duan**

Supporting Information

Chiral Luminescent Liquid Crystal with Multi-State-Reversibility: Breakthrough in Advanced Anti-Counterfeiting Materials

*Yonghong Shi, Jianlei Han, Xue Jin, Wangen Miao, Yi Zhang and Pengfei Duan**

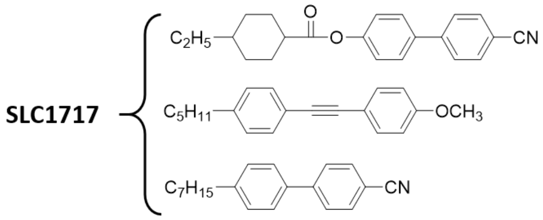

Figure S1. Molecular structure of the components of mixture nematic liquid crystal SLC1717.

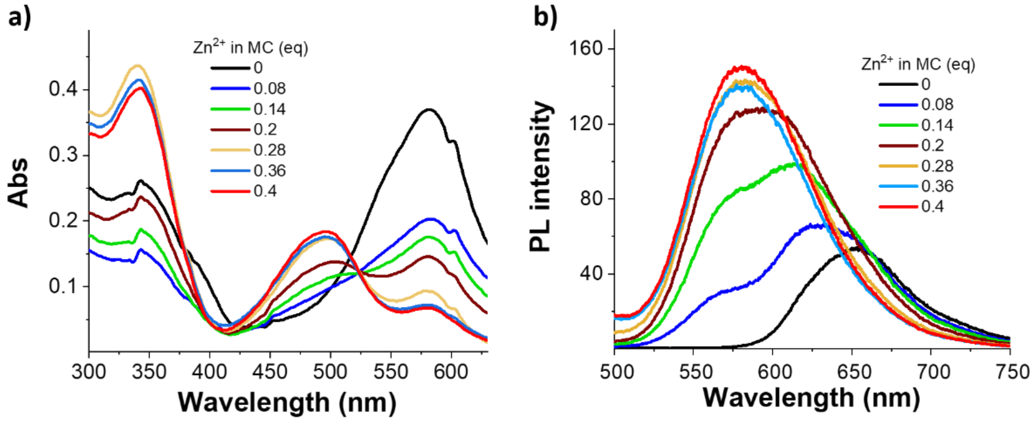

Figure S2. Absorption (a) and emission (b) spectrum (λ_{ex} = 360 nm) of Zn^{2+}/MC with different molar ratios in ethyl acetate (5×10^{-5} M).

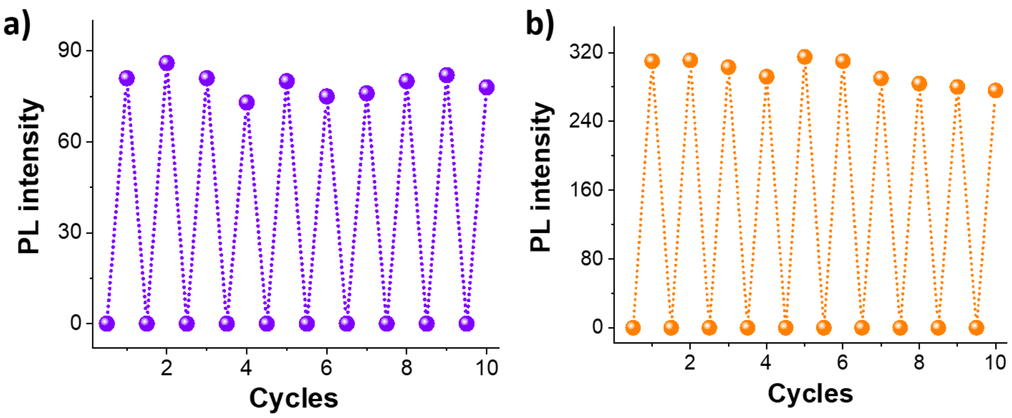

Figure S3. Photoswitching PL intensity of the MC (a) and MC-Zn^{2+} (b) monitored at 650 and 580 nm upon alternating incidence of UV (365 nm) and visible (465 nm) light in ethyl acetate, respectively. ([MC] = [MC-Zn^{2+}] = 5 × 10^{-5} M, λ_{ex} = 390 nm).

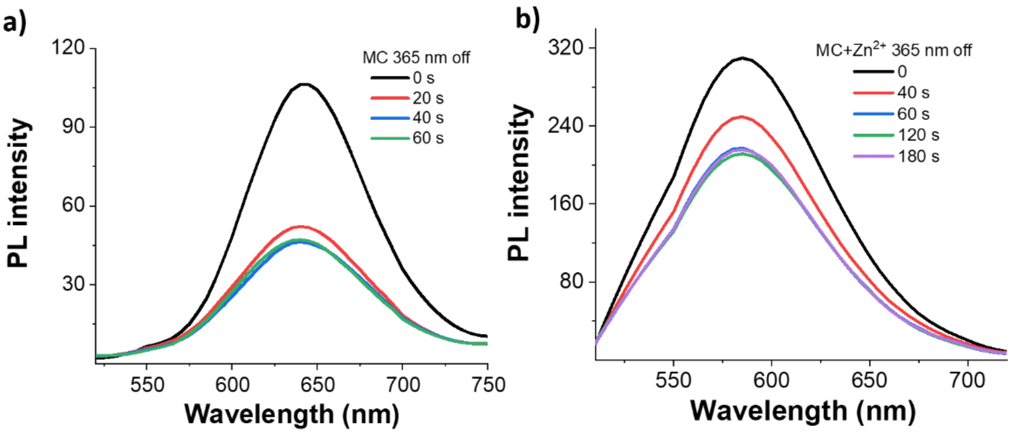

Figure S4. PL intensity decays of the MC (a) and MC-Zn^{2+} (b) with time in ethyl acetate ([MC] = [MC-Zn^{2+}] = 5 × 10^{-5} M, λ_{ex} = 390 nm).

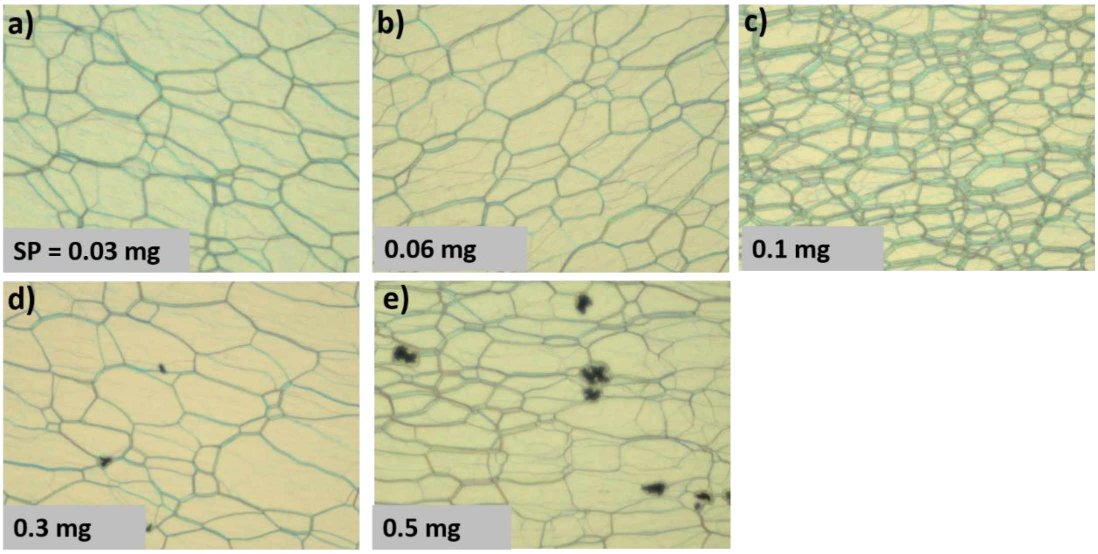

Figure S5. Planar texture of SP/SLC1717 with different weight ratio in LC (*S*811 = 3 mg, SLC1717 = 10 mg).

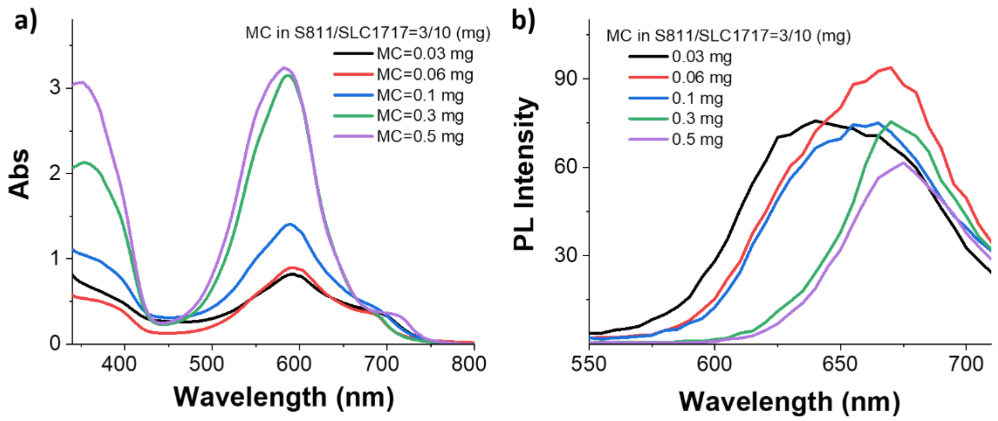

Figure S6. Absorption **(a)** and fluorescence **(b)** spectrum of MC/SLC1717 with different weight ratio in N*LC (*S*811 = 3 mg, SLC1717 = 10 mg).

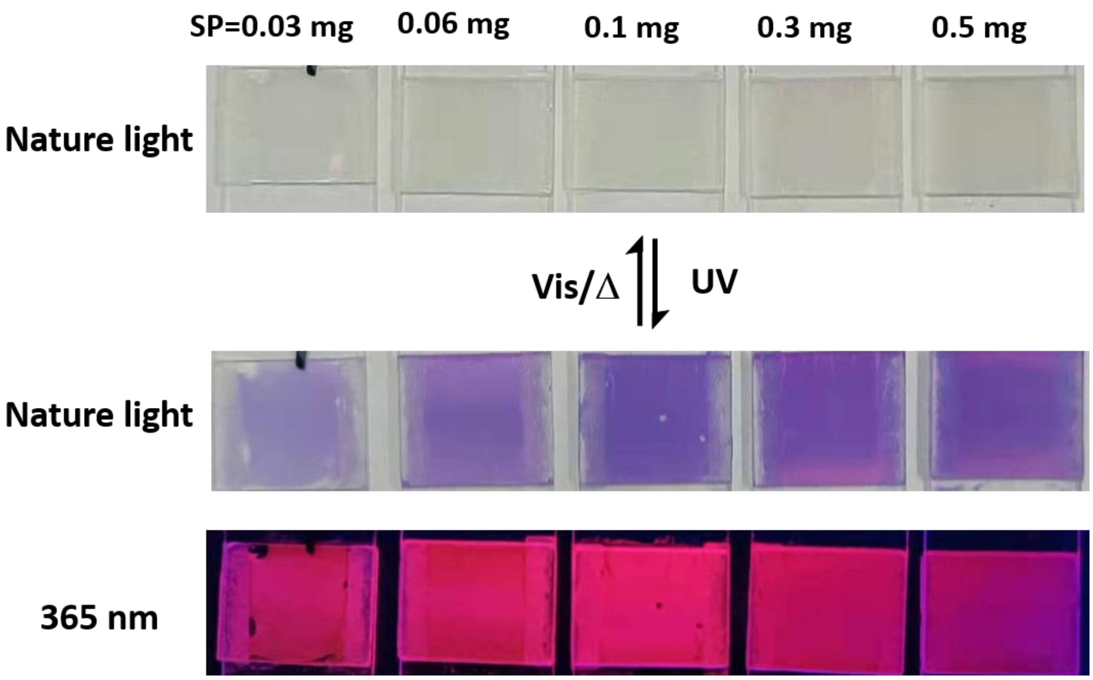

Figure S7. Reversible switching picture of SP/SLC1717 with different weight ratio in N*LC under nature light and UV 365 nm light (*S*811 = 3 mg, SLC1717 = 10 mg).

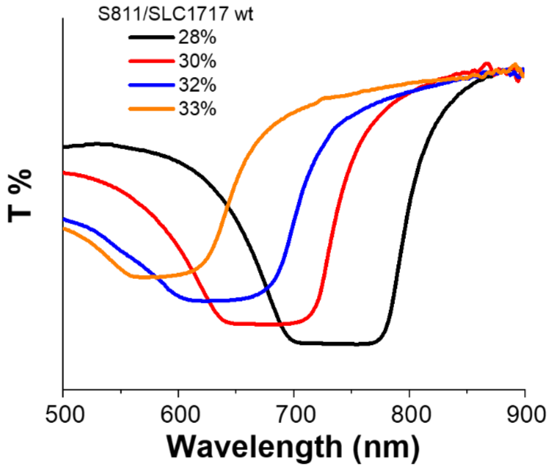

Figure S8. The transmittances spectrum of *S*811/SLC1717 with different weight ratio in LC (SLC1717 = 10 mg, SP = 0.1 mg).

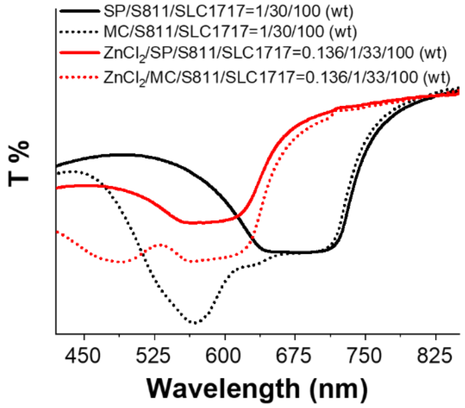

Figure S9. Transmittance spectrum of MC and SP isomer in N*LC system.

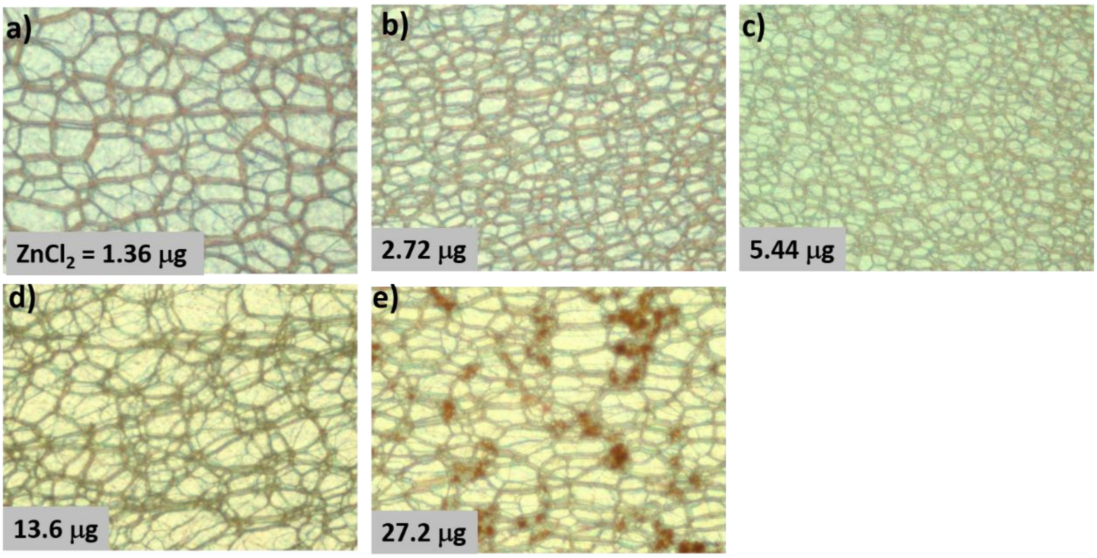

Figure S10. Planar texture of Zn^{2+}/SLC1717 with different weight ratio in SP-N*LC (MC = 0.1

mg, *S*811 = 3.3 mg, SLC1717 = 10 mg).

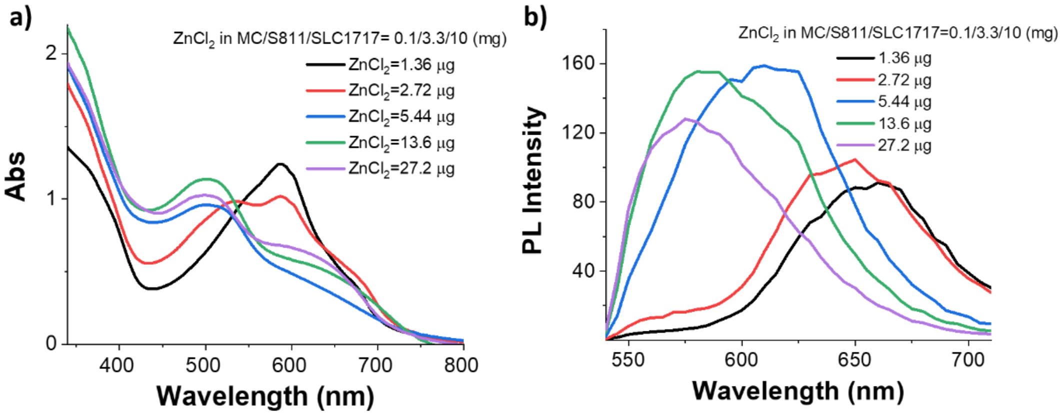

Figure S11. Absorption **(a)** and fluorescence **(b)** spectrum of Zn^{2+}/ SLC1717 with different weight ratio in MC-N*LC (MC = 0.1 mg, *S*811 = 3.3 mg, SLC1717 = 10 mg).

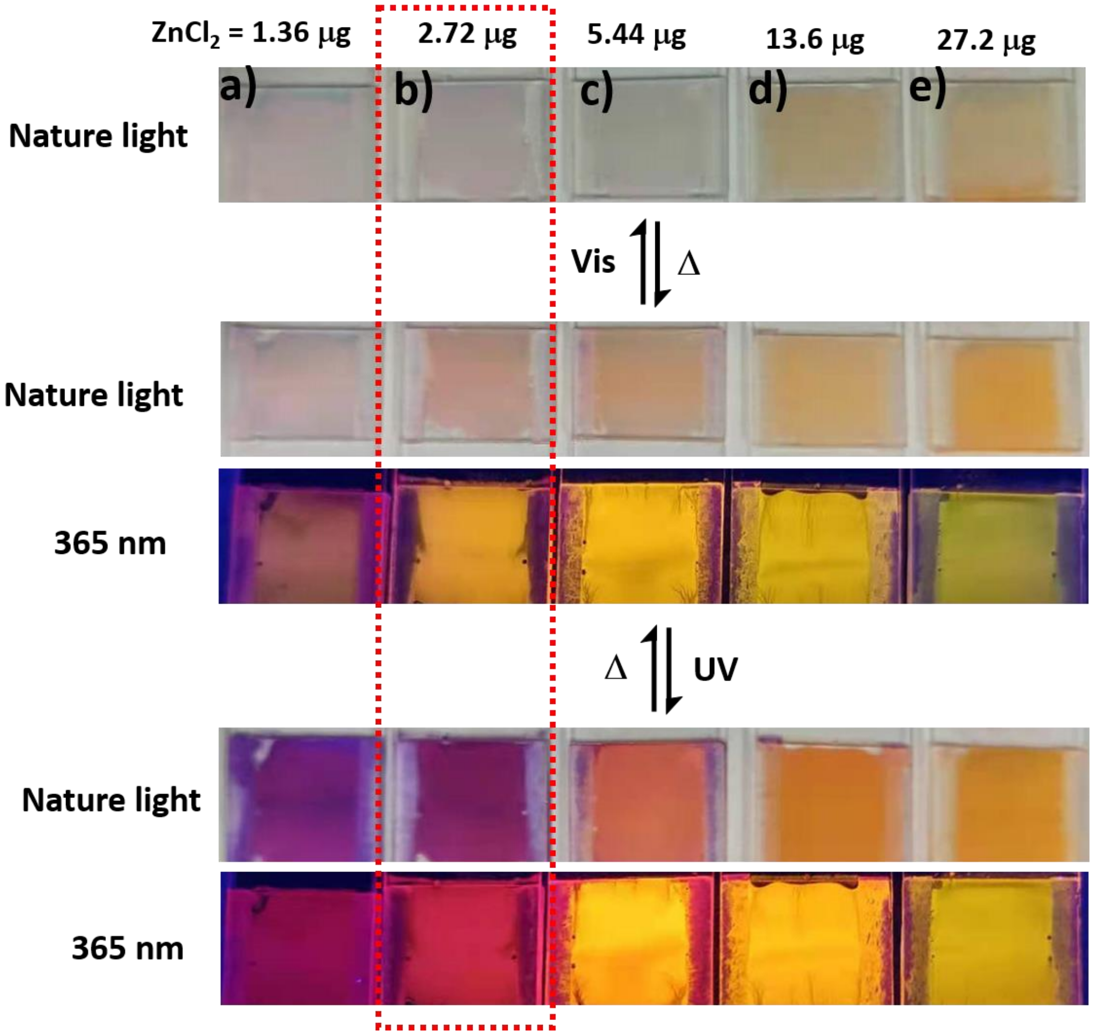

Figure S12. Reversible switching picture of Zn^{2+}/ SLC1717 with different weight ratio in MC-N*LC under UV 365 nm and natural light (SP = 0.1 mg, *S*811 = 3.3 mg, SLC1717 = 10 mg).

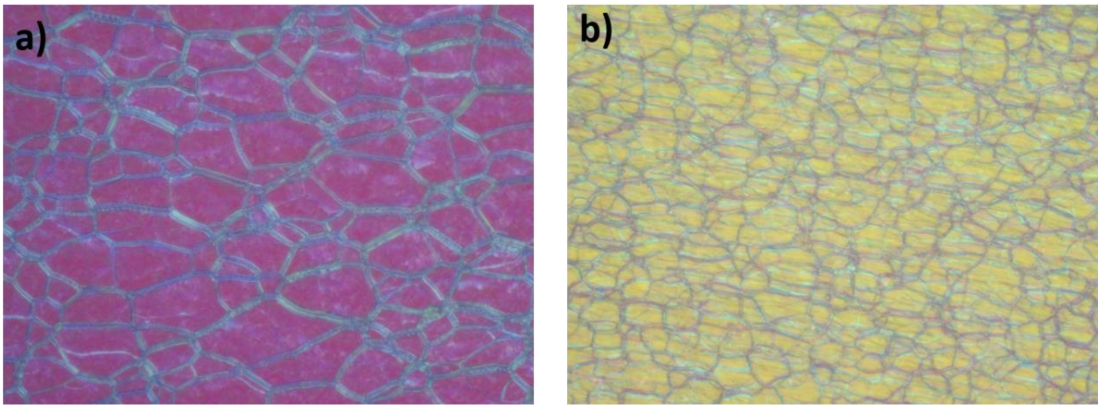

Figure S13. POM images of MC-N*LC (a) and MC-Zn^{2+}-LC (b). The weight ratios of MC/*S*811/SLC1717 = 1/30/100, the weight ratios of $ZnCl_2$/MC/*S*811/SLC1717 = 0.136/1/33/100.

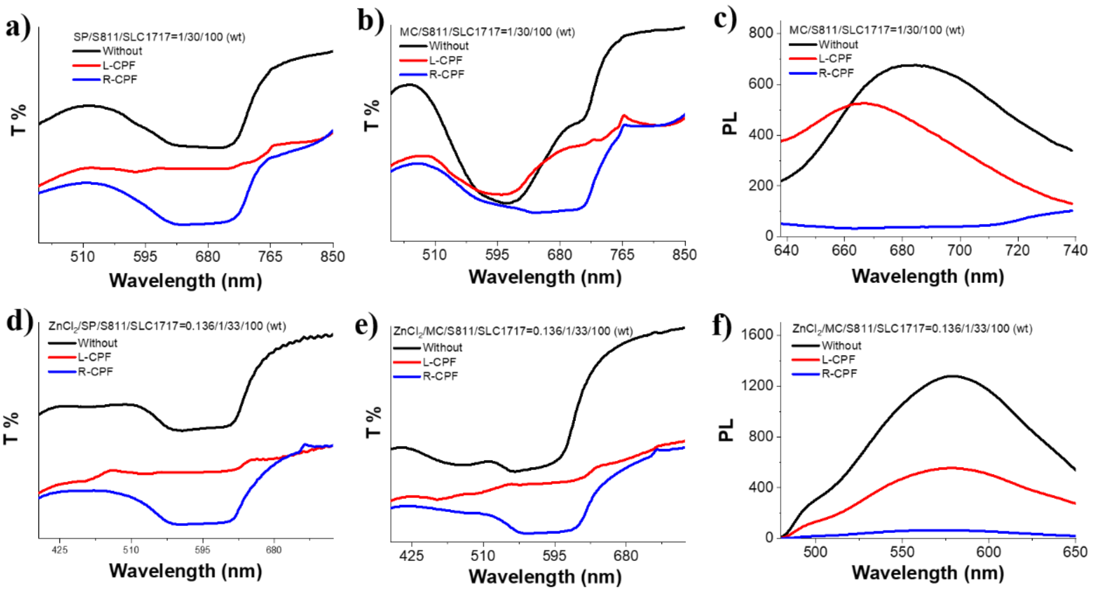

Figure S14. The SP (a) and MC isomer (b) transmittances spectrum and MC isomer fluorescence spectrum (c) in chiral liquid crystal (MC/S811/SLC1717=1/30/100 wt) were tested without CPF, with L- and R-CPF, respectively. The SP/Zn^{2+} (c) and MC- Zn^{2+} isomer (d) transmittances spectrum and MC-Zn^{2+} isomer fluorescence spectrum (e) in chiral liquid crystal ($ZnCl_2$/MC/S811/SLC1717=0.136/1/33/100 wt) were tested without CPF, with L- and R-CPF, respectively.

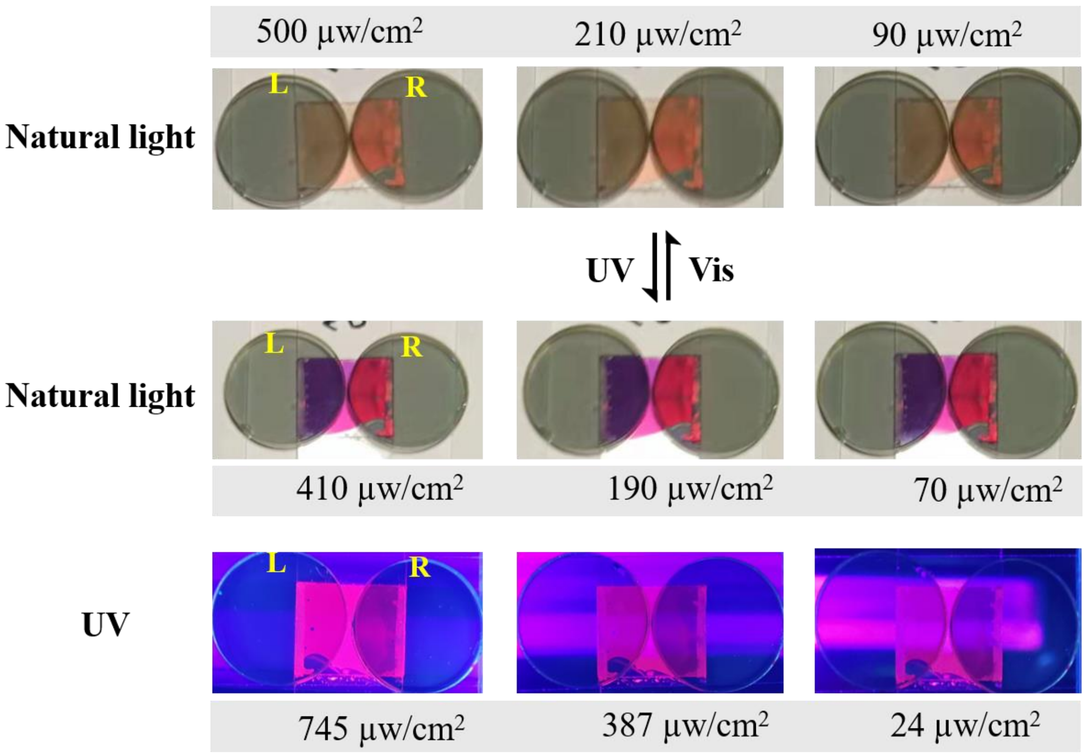

Figure S15. Under different intensities of natural light and UV, the photographs of SP/Zn^{2+}-N*LC and MC-Zn^{2+}-N*LC (S811 and SLC1717) by L-CPF and R-CPF.

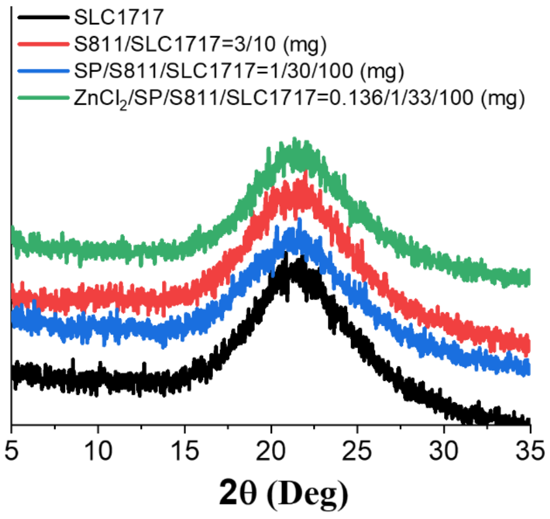

Figure S16. XRD spectra showed that SLC1717 was the non-crystal structure while adding different weight ratios of chiral dopant *S*811 would not influence the structure of liquid crystal. Moreover, the structure of liquid crystal did not change after mixing with different fluorescent molecules and metal ions.

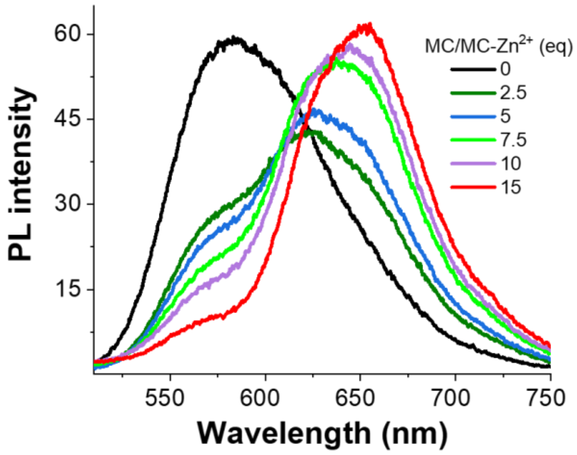

Figure S17. Fluorescence spectral change in ethyl acetate MC-Zn^{2+} induced by MC addition at different molar ratio ([MC-Zn^{2+}] = 10^{-5} M, λ_{ex} = 390 nm). In the experiment, the concentration of MC-Zn^{2+} was kept constant and the concentration of MC was varied.

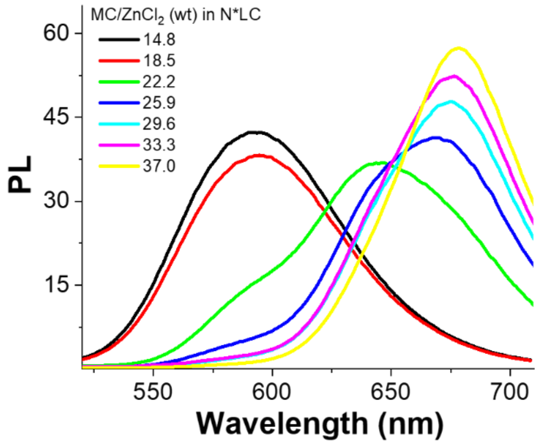

Figure S18. Fluorescence spectral change of MC/MC-Zn^{2+} with different weight ratio in chiral liquid crystal (S811/SLC1717 = 30/100 wt, λ_{ex} = 365 nm). In the experiment, the MC-Zn^{2+} was kept constant ($ZnCl_2$/MC = 14.8 wt, $ZnCl_2$ = 0.027 µg) and the mass ratio of MC was varied.

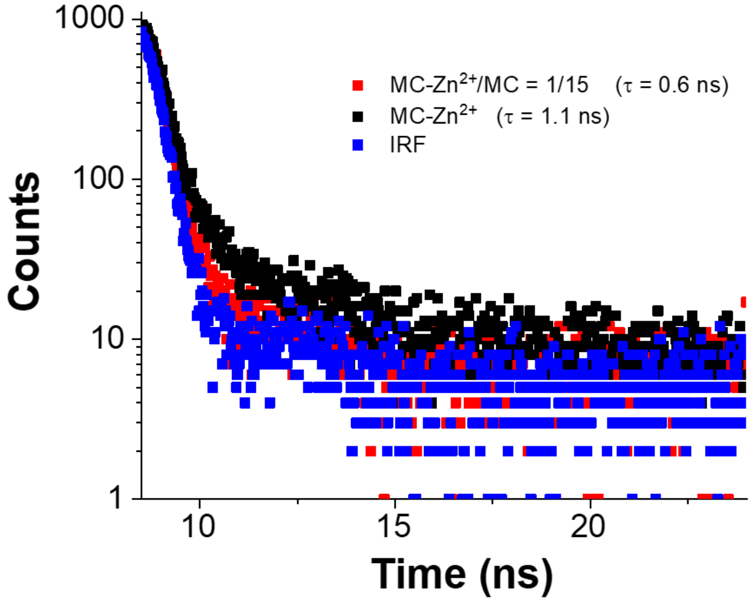

Figure S19. Emission decay curves of the MC-Zn^{2+} and MC/MC-Zn^{2+} = 15/1 monitored at 580 nm (λ_{ex} = 390 nm, [MC-Zn^{2+}] = 10^{-5} M), where the fluorescence lifetime is the average lifetime $\tau = A_1\tau_1 + A_2\tau_2$.

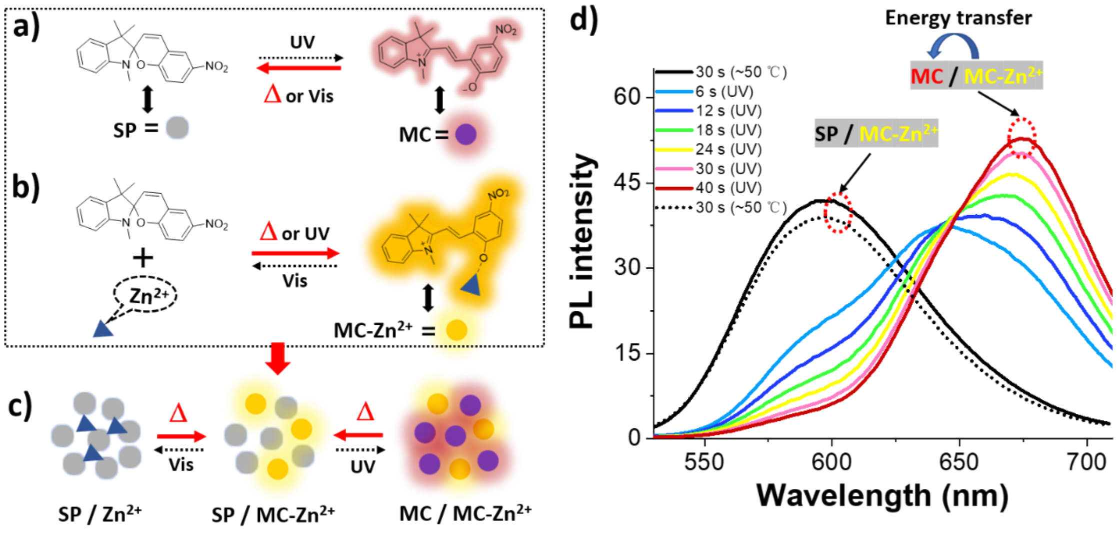

Figure S20. a, b) Reversible structural isomerization of MC and MC-Zn^{2+} under different stimuli. c) Reversible structural isomerization of MC/MC-Zn^{2+} (excess fluorescent dye SP and a small amount of Zn^{2+}) under different stimuli. d) Fluorescence spectra of MC/MC-Zn^{2+} in the chiral liquid crystal under different stimuli (heat → UV → heat) ($ZnCl_2$/MC/ *S*811/SLC1717 = 0.027/1/30/100 wt%).

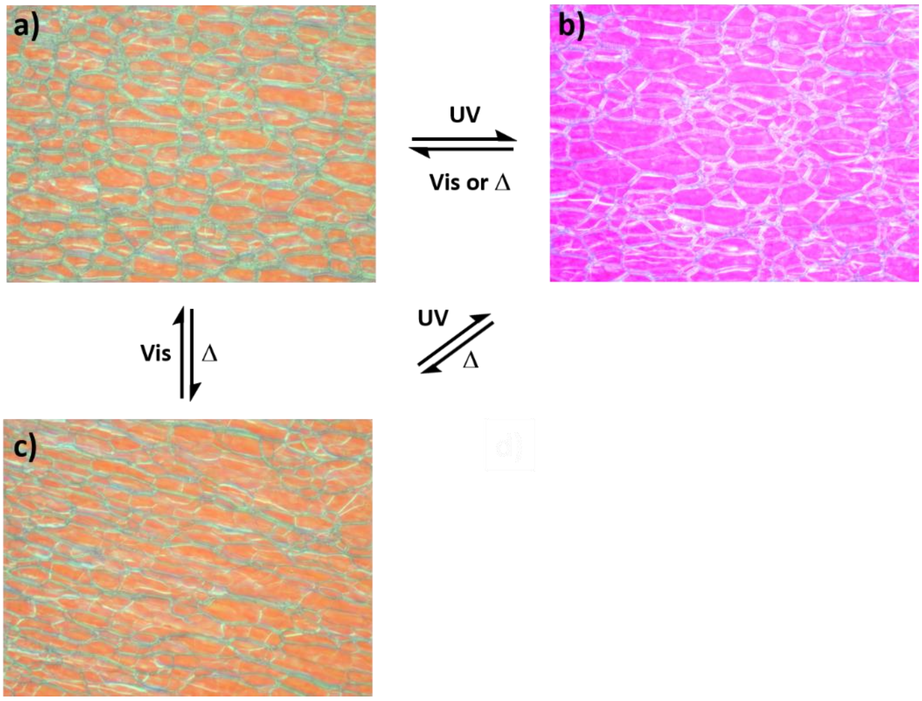

Figure S21. POM images of reversibly multi-states conversion in N*LC ($ZnCl_2$/MC/*S*811/SLC1717 = 0.027/1/30/100 wt%).

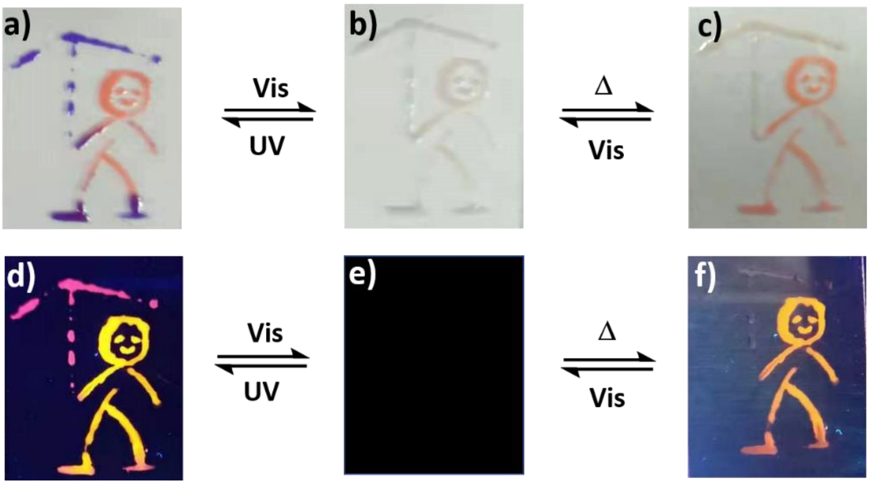

Figure S22. Anti-counterfeiting pattern applications involving the use of the different external simulation responsive (UV, visible and heat) in natural light and UV 365 n light.
